# 5-Methoxyleoligin, a Lignan from Edelweiss, Stimulates CYP26B1-Dependent Angiogenesis In Vitro and Induces Arteriogenesis in Infarcted Rat Hearts In Vivo

**DOI:** 10.1371/journal.pone.0058342

**Published:** 2013-03-12

**Authors:** Barbara Messner, Johann Kern, Dominik Wiedemann, Stefan Schwaiger, Adrian Türkcan, Christian Ploner, Alexander Trockenbacher, Klaus Aumayr, Nikolaos Bonaros, Günther Laufer, Hermann Stuppner, Gerold Untergasser, David Bernhard

**Affiliations:** 1 Cardiac Surgery Research Laboratory, Department of Surgery, Medical University of Vienna, Vienna, Austria; 2 Division of Internal Medicine V, Medical University of Innsbruck, Innsbruck, Austria; 3 Cardiac Surgery Research Laboratory, Department of Cardiac Surgery, Innsbruck Medical University, Innsbruck, Austria; 4 Institute of Pharmacy/Pharmacognosy, University of Innsbruck, Innsbruck, Austria; 5 Department of Plastic and Reconstructive Surgery, Medical University Innsbruck, Innsbruck, Austria; 6 Division Molecular Pathophysiology, Biocenter, Medical University of Innsbruck, Innsbruck, Austria; 7 Department of Pathology, Medical University of Vienna, Vienna, Austria; UCL Institute of Child Health, United Kingdom

## Abstract

**Background:**

Insufficient angiogenesis and arteriogenesis in cardiac tissue after myocardial infarction (MI) is a significant factor hampering the functional recovery of the heart. To overcome this problem we screened for compounds capable of stimulating angiogenesis, and herein investigate the most active molecule, 5-Methoxyleoligin (5ML), in detail.

**Methods and Results:**

5ML potently stimulated endothelial tube formation, angiogenic sprouting, and angiogenesis in a chicken chorioallantoic membrane assay. Further, microarray- and knock down- based analyses revealed that 5ML induces angiogenesis by upregulation of CYP26B1. In an *in vivo* rat MI model 5ML potently increased the number of arterioles in the peri-infarction and infarction area, reduced myocardial muscle loss, and led to a significant increase in LV function (plus 21% 28 days after MI).

**Conclusion:**

The present study shows that 5ML induces CYP26B1-dependent angiogenesis *in vitro*, and arteriogenesis *in vivo*. Whether or not CYP26B1 is relevant for *in vivo* arteriogenesis is not clear at the moment. Importantly, 5ML-induced arteriogenesis *in vivo* makes the compound even more interesting for a post MI therapy. 5ML may constitute the first low molecular weight compound leading to an improvement of myocardial function after MI.

## Introduction

Myocardial infarction (MI) remains a major cause for morbidity and mortality worldwide and is responsible for about one third of all cases of heart failure [1], [2]. Due to the fact that the myocardium has only limited regenerative abilities, the myocardial mass lost as a result of MI is replaced by fibrous tissue. As a compensatory mechanism to the loss of muscular mass, by cardiomyocyte necrosis and apoptosis, the remaining myocardium increases its mass by cardiomyocyte hypertrophy, and tissue remodelling processes (e.g. left ventricular (LV) dilatation). Myocardial remodelling is further based on inflammation, migration and proliferation (e.g of fibroblast) as well as deposition of fibrotic material. Clinical manifested myocardial remodelling could - to some extent-be viewed as useful, but is often not only not sufficient to re-establish cardiac performance, but even contributes to post-MI heart failure [3]. Accordingly, the goal of recent treatment strategies in MI therapy is the induction of “reverse remodeling”, meaning the improvement of ventricular function e.g. by increasing the ejection fraction and the stimulation of angiogenesis and arteriogenesis [4].

Apart from adaptive hypertrophy, (pre- and post-MI) myocardial ischemia also stimulates spontaneous angiogenesis aiming to increase the perfusion of ischemic tissue. This process is mediated by pro-angiogenic cytokines including vascular endothelial growth factor (VEGF) and basic fibroblast growth factor (bFGF). In addition, the recruitment of pericytes and smooth muscle cells (SMCs) is essential in the process [5], [6]. Physiological angiogenesis is however slow (particularly in aged individuals) and the number and size of the new blood vessels is too small to sufficiently supply ischemic regions of the myocardium [7]. Hence, the induction of collateral artery growth to bypass occluded arteries, and consequently improve blood supply of the ischemic areas, is a major scientific goal in the field. In the past years several promising strategies have been tested aiming to accelerate and improve cardiac angiogenesis. Currently the most promising strategies are the injection of growth factors and cytokines (mainly as gene therapies) as well as (stem) cell-based therapies [8], [9], [10], [11], [12]. To date only a few centres successfully apply therapeutic pro-angiogenic treatments in patients. Currently, no strategy is applied on a routine basis, and most of the experimentally successful treatments failed to show a beneficial effect in clinics, indicating the urgent need to develop new strategies and find new drugs. Importantly, none of the above therapeutic options is able to stimulate the essential growth of collateral arteries, and the current opinion in the field is that physical forces (e.g. fluid shear stress) are the primary stimuli for arteriogenesis [13].

## Materials and Methods

### Plant material, isolation, and purification of 5ML

5ML ([(2*S*,3*R*,4*R*)-4-(3,4-dimethoxybenzyl)-2-(3,4,5-trimethoxyphenyl)tetrahydro-furan-3-yl]methyl-(2*Z*)-2-methylbut-2-en-oate) was isolated as previously described from 5.3 kg sub-aerial parts of Edelweiss (*Leontopodium alpinum* Cass.) which were obtained from Swiss horticultures [14]. The purity of the obtained 5ML (78.0 mg) according to LC-DAD/MS- and NMR examination was found to be >98%. Furthermore, a voucher specimen (CH 5002) has been deposited at the herbarium of the Institute of Pharmacy/Pharmacognosy, University of Innsbruck, Innsbruck, Austria.

### Cell culture

Human umbilical vein endothelial cells (HUVECs) and smooth muscle cells were purchased from Promocell (Vienna, Austria) and cultured as previously described [15], [16].

### Wound scratch assay

Migration of SMCs and HUVECs was examined by a wound healing assay. To do so, cells were grown in six-well plates to 80% confluence. A wound was created by scrapping across the surface of each cell monolayer using a pipette tip. Thereafter, the wells were gently washed to remove detached cells followed by addition of culture medium with the indicated concentrations of 5ML. Images of the scratch were taken 6 hours after wound creation with an Olympus microscope (Olympus CKX41, Austria). Dimension of the scratch was analyzed using the Photoshop CS4 software and reduction of the scratch area by migrating cells was calculated. Results are expressed as percent of scratch closure after 6 hours.

### Capillary tube formation assay

To analyze capillary tube-formation, 24-well-plates were coated with 200 µl growth factor reduced Matrigel (BD Biosciences). HUVECs (5×10^4^ cells) were resuspended in 200 µl EGM-2 medium with or without 5ML (1 µM and 10 µM) and placed on the polymerized matrix, followed by the analysis of tube formation after 6 h. Tubes were visualized by an inverted transmission-microscope (Zeiss Axiovert 200 M) and documented by a digital imaging system (Axiovision Software, Zeiss). Statistical analysis was performed after calculating capillaries/mm^2^.

### Spheroid sprouting assay

The assay was performed as described elsewhere [17], with following modifications: HUVEC spheroids where generated overnight in hanging-drop culture consisting of 400 cells in EBM-2 medium, 2% FCS and 20% methylcellulose (Sigma Biochemicals). Spheroids were embedded in collagen type I from rat tail (Becton Dickinson) and stimulated with 50 ng/ml VEGF (Sigma Biochemicals) in the presence or absence of 5ML solution (concentration: 1 µM and 10 µM). Sprouts were also analyzed by inverted transmission-microscopy (Zeiss Axiovert 200 M) and documented by a digital imaging (Axiovision Software, Zeiss). The cumulative sprout length (CSL) was analyzed after printing of high quality pictures and counting by two independent blinded observers.

### Chicken chorioallantoic membrane assay

The chicken chorioallantoic membrane (CAM) assay was used as an established *in vivo* model for screening for pro- and anti-angiogenic proteins and drugs [18]. In brief, fertilized white leghorn chicken eggs (SPF eggs, n = 6 per group) were purchased from Charles River (Kiesslegg) and incubated in an egg incubator at 37°C and 70% humidity (Compact S84, Grumbach) for four days. Subsequently, a window was cut in each eggshell and the underlying membrane. Eggs were incubated for 4 hours with the windows sealed (Durapor™ tape). Then, a Thermanox™ Ring (Nunc) was placed on the CAM and a 10 mM Tris-Glycine solution (pH 7.4) containing 0.1 µg and 0.5 µg 5ML, respectively, was applied to the ring area. For control the pure puffer solution was used. Eggs were sealed and incubated for three days. After removal of the seal, the CAM with the Thermanox™ ring was analyzed and photographed under a stereomicroscope connected to a digital camera and flexible cold light (Olympus SZ51, Olympus E410). Blood vessels were counted in the ring area (20 mm^2^).

### Microarray analyses

After incubation of primary HUVECs for 6 or 24 h with 10 µM of 5ML, cells were detached by trypsinisation, and collected by centrifugation (300 g, RT, 5′). Cell pellets were stored at -20°C. For RNA preparation, the pellets were resuspended in TriReagent (Sigma) and RNA was isolated as previously described [19], [20]. Total RNA was further purified using the RNeasy kit (Qiagen, Hilden, Germany). RNA quantity and integrity were assessed by OD260/280 measurements and Agilent “lab-on-a-chip” technology (2100 Bioanalyzer, Agilent, Palo Alto, CA). Quality requirements were OD260/280 ratios of 1.8 to 2.2 and an 18S to 28S ratio of 1.8 to 2.2. Hybridization target preparations were performed according to protocols recommended by Affymetrix (Affymetrix Technical Manual, protocol 2). Briefly, 5 µg total RNA was reverse transcribed using an oligo(dT)-T7 promotor primer, before second strand synthesis by E.coli DNA polymerase (Affymetrix one cycle cDNA synthesis kit). After purification of double stranded cDNA with the Affymetrix GeneChip Sample Cleanup Module, biotin-labeled cRNA was produced by T7 polymerase (Affymetrix IVT Labeling kit). After lab on a chip-based quantification and integrity control, 20 µg cRNA was fragmented by alkaline treatment (Affymetrix GeneChip Sample Cleanup Module), and 15 µg fragmented cRNA was added to the Affymetrix hybridization cocktail (300 µl final volume). The arrays (Human genome U133 Plus 2.0; Affymetrix) were washed and stained according to the recommended Fluidics Station protocol (EukGE-WS2 version 5_450). Fluorescence signal intensities from each feature on the microarrays were determined using the Affymetrix GeneChip Scanner 3000 and GCOS software (version 1.2), according to the manufacturer's recommendations. The raw data from all arrays were normalized using the RMA package for “R” according to [21]. Microarray analyses were performed on three independent experiments, and the genes reported to be regulated were found to be regulated in all experiments. Genes with an expression value A below 5 were excluded from further analysis. Genes were considered to be regulated when the log_2_ ratio of the expression values (M) was identical to or below -1 (two-fold down-regulation) or when M was identical to or above 1 (two-fold up-regulation).

### Knock down of CYP1A1 and CYP26B1

SiRNA-mediated knock down of CYP1A1 and CYP26B1 was performed using customised siRNAs (Santa Cruz Biotechnology, Santa Cruz, CA, USA) and the Amaxa Nucleofector (Lonza Group, Basel, Switzerland) as described by the manufacturers (also see [22]). For the knock down of CYP1A1 and CYP26B1 HUVECs were transfected with CYP1A1 siRNA, CYP26B1 siRNA, or control oligos according to the manufacturer's instructions. Knock downs were verified by Western blotting: primary antibodies anti-CYP1A1 (Biomol, Germany); anti-CYP26B1 antibody (Abcam, UK). For protein loading control membranes were stained with Ponceau-S. Quantification of bands was carried out using Quantity One 4.6.1 1-D Analysis software (Bio-Rad, Hercules, CA, USA)). Transfected cells were consequently subjected to the treatments and analyses indicated.

### Animal model of MI

Ethics Statement: All animals received care in compliance with the ‘Principles of laboratory animal care’ formulated by the National Society for Medical Research and the ‘Guide for the care and use of laboratory animals’, prepared by the Institute of Laboratory Animal Resource and published by the NIH. This study was approved by the Austrian Ministry of Science and Research. The authors of this manuscript have certified that they comply with the Principles of Ethical Publishing in the International Journal of Cardiology [23]. Male Wistar rats weighing 250–300 g underwent induction of MI by ligation of the left anterior descending artery (LAD). Animals were anesthetized by intra muscular injection of a combination of ketamine (100 mg/kg) and xylazine (10 mg/kg), and were ventilated after orotracheal intubation. Quality of intra-operative anesthesia was assessed by heart rate measurements and pain response to forceps pinch in the toe region. After a left minithoracotomy, the pericardium was opened, and the proximal LAD was ligated with Prolene 7–0 sutures to induce a sizable infarct. Using a 27 g needle, 30 minutes after ligation of the LAD solvent control or a 10 µM 5ML solution was injected into the peri-infarction zone (5 injections à 10 µl per animal), followed by closure of the operation situs. The infarction area was identified by its white color; the peri-infarction area was defined as a 1 mm thick ring around the infarction area. Correct application of the solutions was ensured by 1 mm depth of injection, control by aspiration, and the formation of epicardial “bubbles” on the surface after injection.

Preparation of solutions for injection: 5ML was dissolved in DMSO giving a 100 mM solution. This solution was then dissolved in 0.9% NaCl solution to give a final concentration of 10 µM, which was used for injections. The control solution was generated exactly the same way using DMSO without 5ML.

### Analysis of myocardial function

Echocardiographic studies were performed with a high-frequency linear array transducer (SONOS 5500, Hewlett Packard, Andover, Mass). Two-dimensional images were obtained at midpapillary and apical levels. End-diastolic (EDV) and end-systolic (ESV) LV volumes were obtained by biplane area-length method, and percent LV ejection fraction was calculated as ([EDV-ESV]/EDV) x 100. Echocardiographic analyses were performed at day 1 prior to surgery (baseline) and days 1, 14, and 28 after MI. Myocardial function was assessed in anaesthetized animals (anesthesia as above).

### Animal sacrification and preparation for morphological studies

Rats were euthanatized, hearts were first arrested in diastole using a 1 M cardioplegic potassiumchloride (KCL) solution and then harvested, fibrous tissue was removed and after rinsing the intracardiac blood, hearts were divided into two equally thick parts representing the base and the apex of the heart.

### Immunohistological and histological analyses

Following fixation in 4% paraformaldehyde and dehydration of heart tissues, tissues were embedded in paraffin and cross sections were prepared. After deparaffinization, histochemical stainings were performed using the Masson Trichrome staining kit (Merck, Germany) as described by the manufacturer. Image acquisition was conducted with Aperio Scan Scope CS and for image-analysis Photoshop CS4 software was used. Evaluation of MI area was conducted by quantification of the fibrotic area (stained in blue, viable heart muscle is stained in red) and calculated as the percentage of the whole myocardial area. The peri-infarction area was defined as boarder zone extending the infarction area between 0.8 and 1.2 mm (area depending on size of infarction). The viable heart muscle tissue (clearly visible as red staining after Massońs Trichrome staining) was also analyzed with Photoshop CS4 and calculated as area within the whole fibrotic infarction area. Additional, a histochemical staining was performed using haematoxylin/eosin (HE) according to the manufacturerś instructions to analyse and count the number of arterioles (counted arterioles were divided into three main groups: arterioles with 2–3 smooth muscle cell layers; small arteries with 3–8 smooth muscle layers, and arteries with more than 8 smooth muscle layers) and capillaries in the defined infarction and per-infarction areas. The analysis of septum thickness was performed using the Aperio ImageScope software. The thickness of the cardiac septum was measured at ten different points of the HE-stained heart sections and calculated as the ratio of septum thickness to the total heart diameter.

To quantify the expression of CYP26B1 and Ki67, standard deparaffinisation and heat-mediated antigen retrieval in sodium-citrate buffer were performed. After blocking in 2% BSA, sections were incubated with anti-CYP26B1 antibody (Abnova, Taiwan), or anti-Ki67 antibody (Abcam, Cambridge), rinsed, and incubated with HRP labeled secondary antibody as described by the manufacturer (ABC-Kit anti-goat, Vectasatin PK-4005; or ABC-Kit universal anti-rabbit/mouse, Vectastain PK-6200; Substrate-Kit for peroxidase activity, Vector Lab. SK-4100). After washing, sections were mounted, and acquired using AxioVision Rel. 4.8 software (Zeiss, Oberkochen, Germany). CYP26B1- and Ki67- positive cells were counted. Results are expressed as number of cells in the whole infarction area. Image analysis was conducted by two independent blinded researchers.

### Tunel assay

TUNEL staining was performed on histological sections of hearts (5ML treatment and controls) using the In Situ Cell Death Detection Kit (POD, Roche; Cat.No. 11684817910). In brief, 5 µm sections were deparaffinised in a graded ethanol series (Xylol, 100% ethanol, 96% ethanol, 70% ethanol) and permeabilized with 0.1% triton in 0.1% sodium-citrate buffer. Following 3 washing steps in PBS, the nuclei were stained with propidium iodide (50 µg/ml) for 1 min. Labeling of DNA strand breaks was performed according to the manufacturer's instructions. Sections were analyzed using a LSM510 Meta attached to an Axioplan 2 imaging MOT using ZEN software. Quantification of assay results was performed by three independent researchers by counting the tunnel-positive cells per total cell count (propidium iodide positive cells).

### Statistical analyses

Where indicated data were analyzed for a Gaussian distribution and consequently subjected to parametric tests (one way ANOVA (Bonferroni adjusted) or two-sided t-test) or non-parametric tests (Mann-Whitney U Test).

## Results

We previously conducted a screening approach set to find novel angiogenesis modulatory compounds. This screen was based on the analysis of natural and chemically synthesized derivatives of the novel structure type lignan Leoligin [14], [16], [24], that was previously isolated from the roots of Edelweiss (*Leontopodium alpinum* Cass.) by our team. In this approach 5ML showed promising pro-angiogenic effects and was therefore subjected to an in depth analysis for its angiogenesis- and arteriogenesis- promoting properties in the present study. This approach was chosen, as low molecular weight (hydrophobic) pharmaceuticals-in contrast to nucleic acids, peptides, proteins or even cells-are capable of easily penetrating cellular membranes and tissues.

### 5ML is a stimulator of capillary tube formation, angiogenic sprouting *in vitro*, and of angiogenesis in a chicken chorioallantoic membrane assay

**Figure 1:**
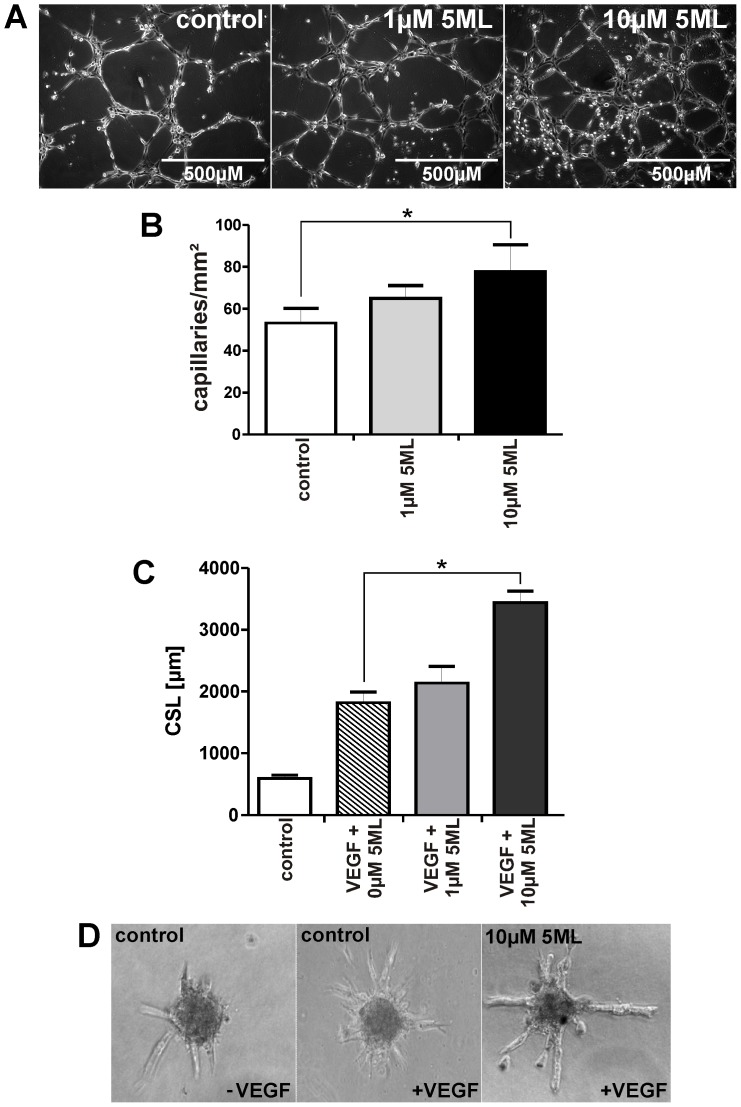
5ML promotes capillary tube formation and angiogenic sprouting of human endothelial cells in a dose dependent manner. Figure 1A: Effects of increasing concentrations of 5ML on human endothelial cells were analyzed in a capillary tube formation assay in matrigel (magnification: 100×). Figure 1B: Numbers of capillaries per mm^2^ were determined 6 hours after incubation with 1 and 10 µM 5ML. Figure 1C: Spheroids of HUVECs were stimulated with 50 ng/mL VEGF and different concentrations of 5ML or DMSO as solvent control. Cumulative sprout length (CSL) was analyzed 24 hours after stimulation (Figure 1D). All experiments were performed in triplicates. Asterisks indicate significant differences (* p<0.05) compared to the corresponding controls.

**Figure 2:**
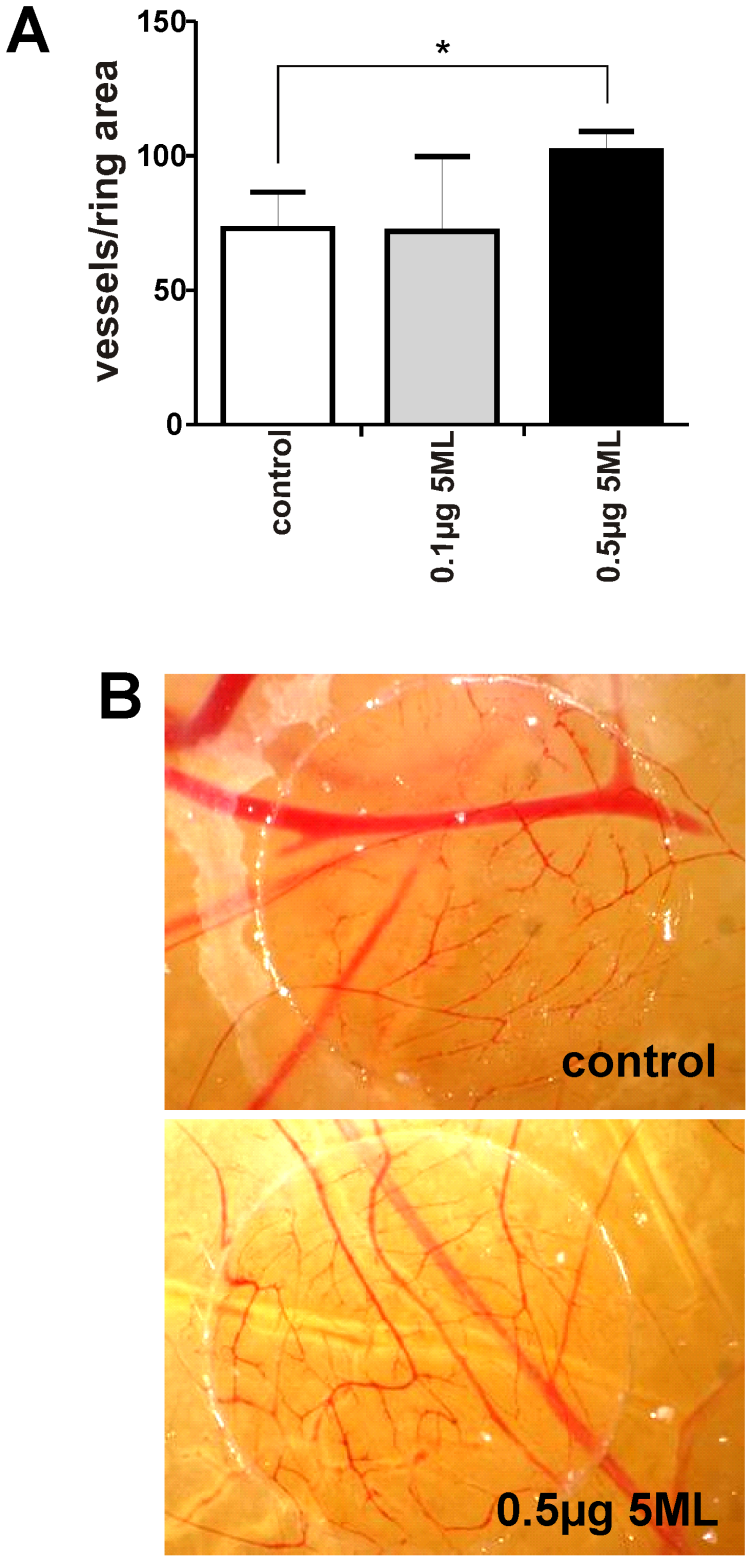
Effects of 5ML on blood vessel formation in the chicken chorioallantoic membrane assay (CAM) assay. Figure 2A: CAMs of 3-days- old chickens were incubated for 96 hours with the control solution (DMSO as the solvent control) or 0.1 or 0.5 µg 5ML. Figure 2A shows that the treatment with 5ML enhanced the formation of new blood vessels. 5ML significantly increased blood vessel formation at a concentration of 0.5 µg/egg. Figure 2B shows representative images of the CAM-assay (Magnification: 12,5×). Blood vessels were counted in the ring area of the CAM to quantify effects of 5ML *in vivo*. All experiments were performed in triplicates. Asterisks indicate significant differences (* p<0.05) compared to the corresponding controls.

In order to analyze the angiogenesis-stimulatory capacity of 5ML, human umbilical vein endothelial cells (HUVECs) were treated with 5ML and tube formation was analyzed. A dose-dependent and significant increase in tube formation was observed by 5ML treatment (Figure 1A and 1B). The same analyses were performed on human microvascular endothelial cells (HMVECs) giving similar results (Supporting Information file, Figure S3). To confirm these data we also analyzed endothelial spheroid sprouting in the presence and absence of 5ML. In this assay 5ML induced a significant increase in spheroid sprouting (Figure 1C and 1D). As the next step the angiogenesis-promoting activity of 5ML was measured in an *in vivo* chicken chorioallantoic membrane assay. The data in Figure 2 show that 5ML led to a significant increase in capillary density in the chicken chorioallantoic membrane (Figure 2A and 2B).

### 5ML stimulates angiogenesis by increasing the expression of CYP26B1

**Figure 3:**
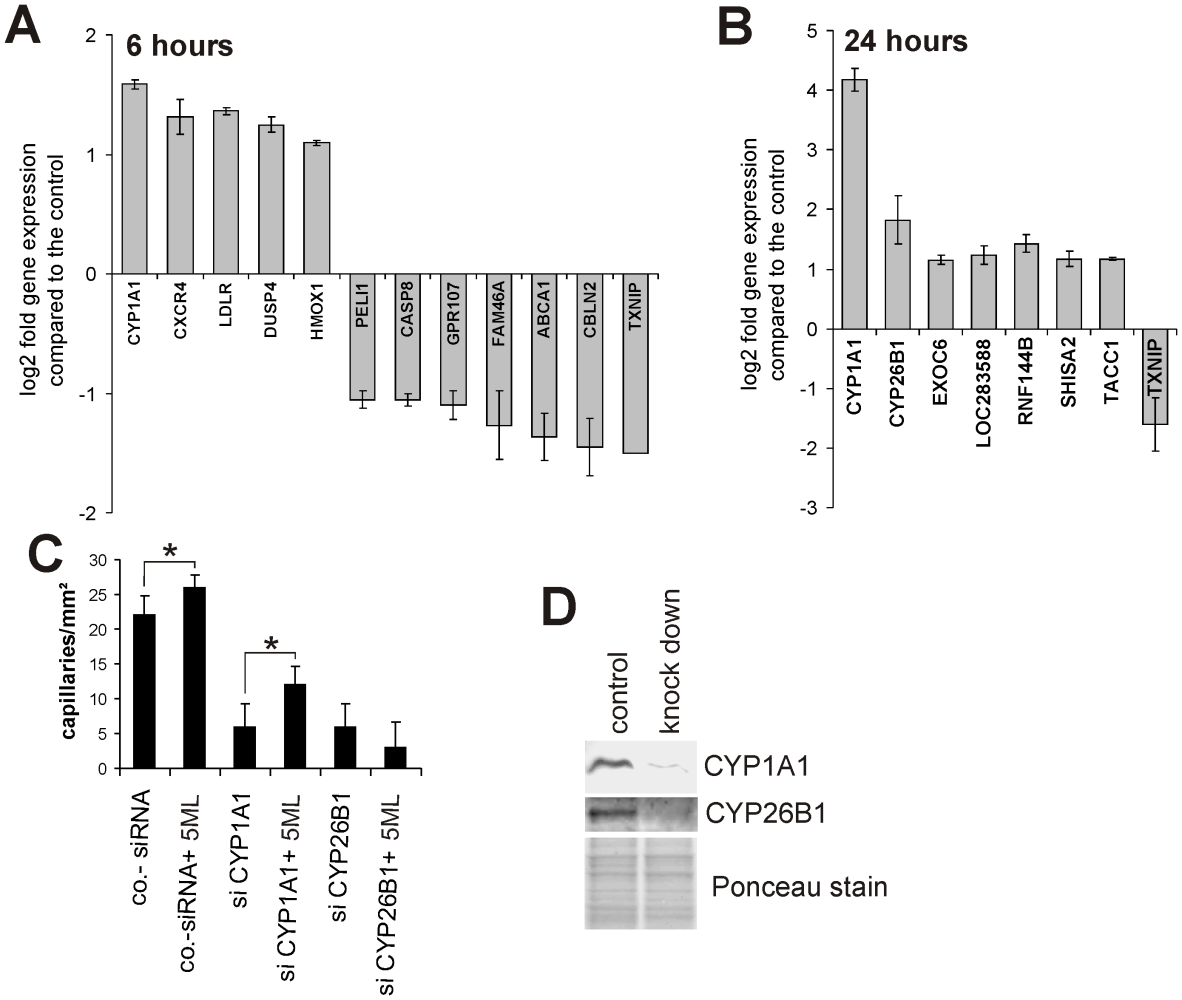
5ML alters the transcription of a small set of genes; CYP26B1 is the causal mediator of 5ML -induced angiogenesis. Figures 3A and 3B show median values +/− S.D. of microarray-based analyses of gene-regulation by 5ML in relation to controls 6 and 24 hours after the addition of 5ML (10 µM). Figure 3C shows that despite the fact that the knockdown of CYP1A1 had a significant inhibitory effect on spontaneous angiogenesis in HUVECs; CYP1A1 knockdown had no effect on angiogenesis increased by 5ML. Similarly, CYP26B1 knockdown potently repressed spontaneous angiogenesis in HUVECS and in addition completely abrogated 5ML-induced tube formation. Shown are mean values of two independent experiments performed in triplicates +/− SD. * p<0.05. Figure 3D shows Western blot analyses of effects of the CYP1A1- and the CYP26B1-specific knockdown on protein expression of the corresponding proteins. Asterisks indicate significant differences (* p<0.05) compared to the corresponding controls.

In order to reveal genes that might be involved in 5ML-induced angiogenesis we performed microarray-based expression analyses of HUVECs in the presence or absence of 10 µM 5ML for 6 and 24 hours. Figure 3A and 3B show that cytochrome P450 1A1 (CYP1A1) is the most highly upregulated gene and that thioredoxin interacting protein (TXNIP) is the most downregulated gene after 6 and 24 hours of exposure to 10 µM 5ML. Based on these results we performed functional analyses by 5ML treatment of CYP1A1 knock down (siRNA-mediated) and TXNIP over-expressing (lentivirus mediated) HUVECs followed by capillary tube formation assays. Interestingly, CYP1A1 knock down had a dramatic effect on spontaneous (i.e. 5ML independent) tube formation (see Figure 3C), however, like TXNIP over-expression (which had no effect on spontaneous tube formation-see Figure S1 in the Supporting Information file), no significant effect on 5ML-induced increase in tube formation was observed. Therefore we expanded the knock down and over-expression approach to other genes found to be regulated by 5ML. As shown in Figure 3C, siRNA mediated knock down of CYP26B1 totally abrogated 5ML-mediated increase in capillary tube formation. Western blot based analysis were performed to verify the knock down of CYP1A1 and CYP26B1 protein expression (Figure 3D; ponceau staining served as loading control).

### 5ML significantly increases cardiac performance, reduces the septum diameter but has no influence on the size of the infarction area of rat hearts after MI

**Figure 4:**
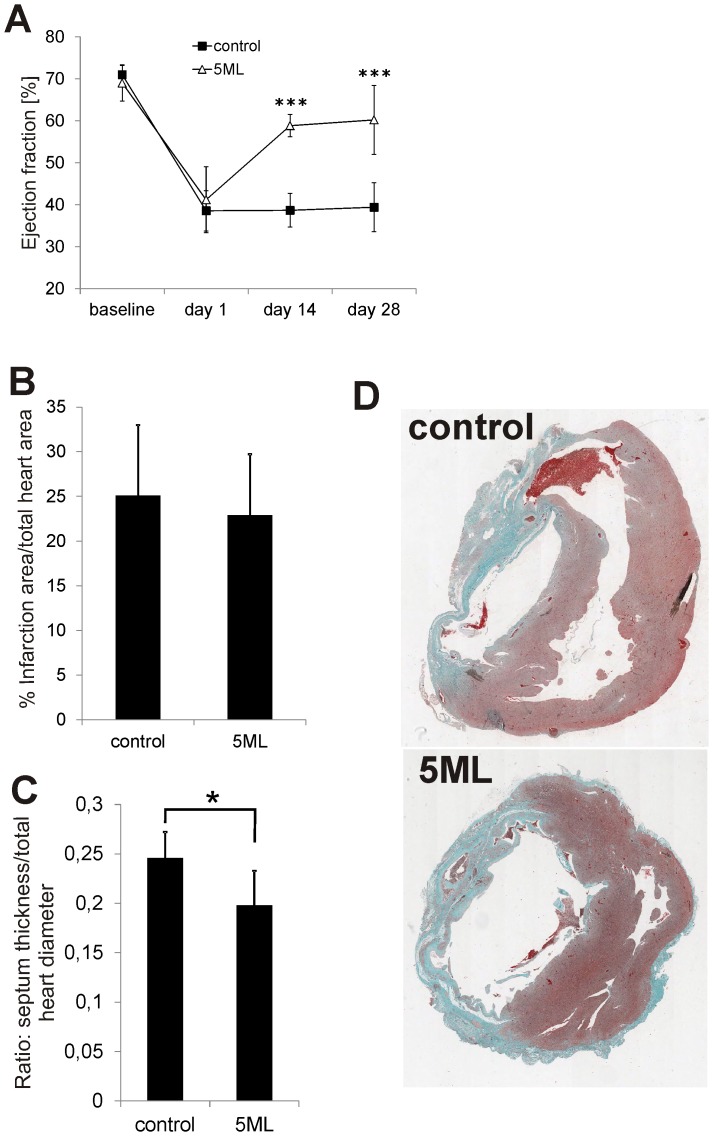
5ML treatment improves the ejection fraction, inhibits cardiac hypertrophy, but has no effect on the infarction size in rats after MI. Figure 4A shows data from an ultrasound-based analysis of the LV ejection fraction in rats before (baseline) and after MI (day 1, 14 and 28 post MI), either after the injection of the solvent control or the injection of 5ML. Shown are mean values +/− SD of all animals (n = 7 per group). Figure 4B shows the quantitative analysis of the infarction size (shown as% of total heart size) of control and 5ML treated rat hearts (n = 7 per group). The septum diameter of control and 5ML treated (n = 7 per group) rat hearts was analyzed and results are depicted in Figure 4C. Representative images of Masson Trichrome stained control and 5ML treated heart sections are shown in Figure 4D (magnification 20×). Asterisks indicate significant differences (* p<0.05; *** p<0.001) compared to the corresponding control group.

In order to expand our previous findings in the chicken chorioallantoic membrane assay towards a more clinical setting, we induced MI in rats by ligature of the left anterior descending artery (LAD). After infarction either solvent control or 10 µM of 5ML was injected into the peri-infarction zone. Prior to surgery (baseline) and on days 1, 14, and 28 days after infarction the LV ejection fraction (EF) of the rat hearts was analyzed by ultrasound. Figure 4A shows that in the 5ML group the absolute EF increased statistically highly significant by 21% compared to the control group. Nevertheless, histological analyses gave that there is no significant difference in infarct size between the groups (control: 25.08% infarction size per total heart size; 5ML: 22.90% infarction size per total heart size; p-value: 0.609; Figure 4B and 4D). However, control group hearts showed signs of hypertrophy, indicated as an increased septum thickness compared to the 5ML treated rat hearts (p = 0.017 Figures 4C).

### 5ML protects the myocardium, induces increased CYP26B1 expression in the infarction area and reduces apoptosis of cardiomyocytes in the peri-infarction area

**Figure 5:**
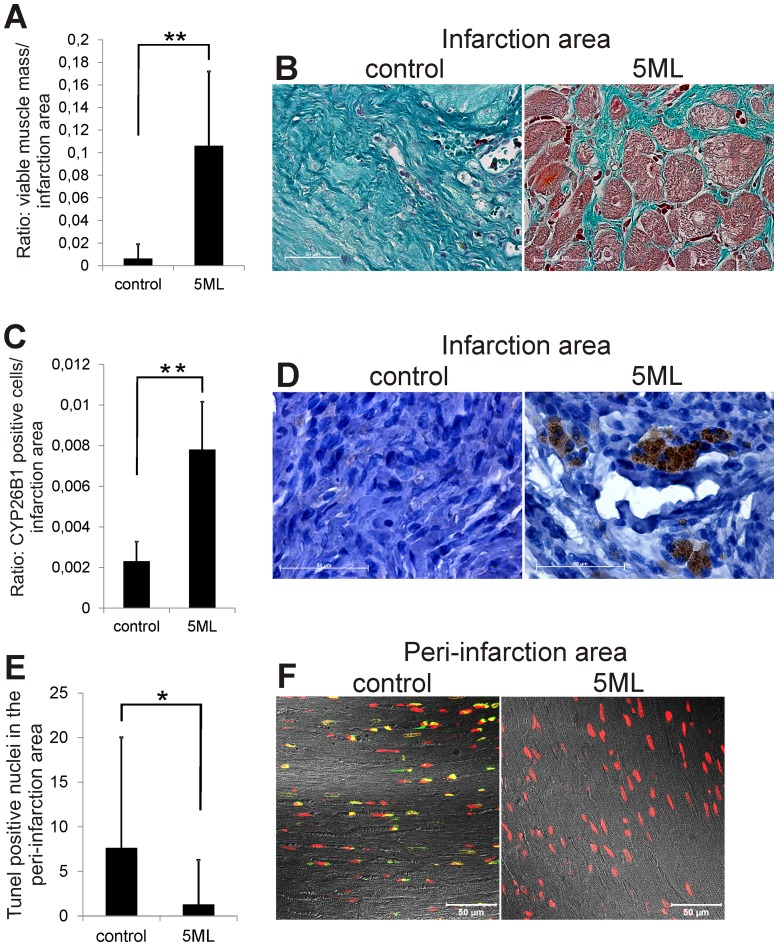
5ML treatment increase the amount of viable cardiac tissue, increases the CYP26B1 expression in the infarction area, and reduces the number of Tunel positive cells in the peri-infarction area. Figure 5A shows the quantification of the viable cardiac tissue within the highly fibrotic infarction area (n = 7 per group). Figure 5B shows the staining of heart sections with Masson's Trichrome that visualizes the viable heart muscle (red staining) and the fibrotic area (intensive blue staining) (magnification 63×). Quantitative image analysis (of all images, n = 7 per group) of the CYP26B1 expression in the infarction area is depicted in Figure 5C. Figure 5D shows representative images of CYP26B1-immunohistochemically stained heart sections of control and 5ML treated rat hearts (magnification 100×). Quantification of the number of Tunel positive nuclei in the peri-infarction area is shown in Figure 5E (n = 7 per group). Figure 5F shows representative images of Tunel-stained cardiomyocytes in the per-infarction area of control and 5ML treated rat hearts (magnification 40×). Asterisks indicate significant differences (* p<0.05; ** p<0.01) compared to the corresponding control group.

Additional histological analysis revealed that 5ML induces processes which are able to contribute to the improvement of cardiac performance. First, treatment of the infracted myocardium with 5ML protected from loss of viable muscle mass within the infarction area (Figure 5A): staining of heart sections with Massońs Trichrome (Figure 5B) showed the presence of viable heart muscle (demonstrated by the red staining) within the fibrotic infarction area of treated rat hearts, but not in controls. Secondly, as the *in vitro* experiments identified CYP26B1 as the central player in 5ML induced angiogenesis, the analysis of the *in vivo* myocardial expression of CYP26B1 also showed a significant increase in CYP26B1 expression (Figure 5C and 5D). Interestingly, the expression of CYP26B1 was limited to the infarction area and was essentially missing in the viable heart muscle of both groups. Thirdly, 5ML induced protection of cardiomyocytes was not only limited to the infarction area. Analysis of DNA-strand breaks as the result of apoptotic signaling in cardiomyocytes (verified by the overlay of fluorescence images with transmitted light images of the peri-infarction area) indicated that 5ML significantly reduced the number of TUNEL positive cells also in the peri-infarction area (Figure 5E and Figure 5F).

### 5ML induces arteriogenesis but not angiogenesis in the infarction area of rat hearts after MI and induces the migration of endothelial and smooth muscle cells in vitro

**Figure 6:**
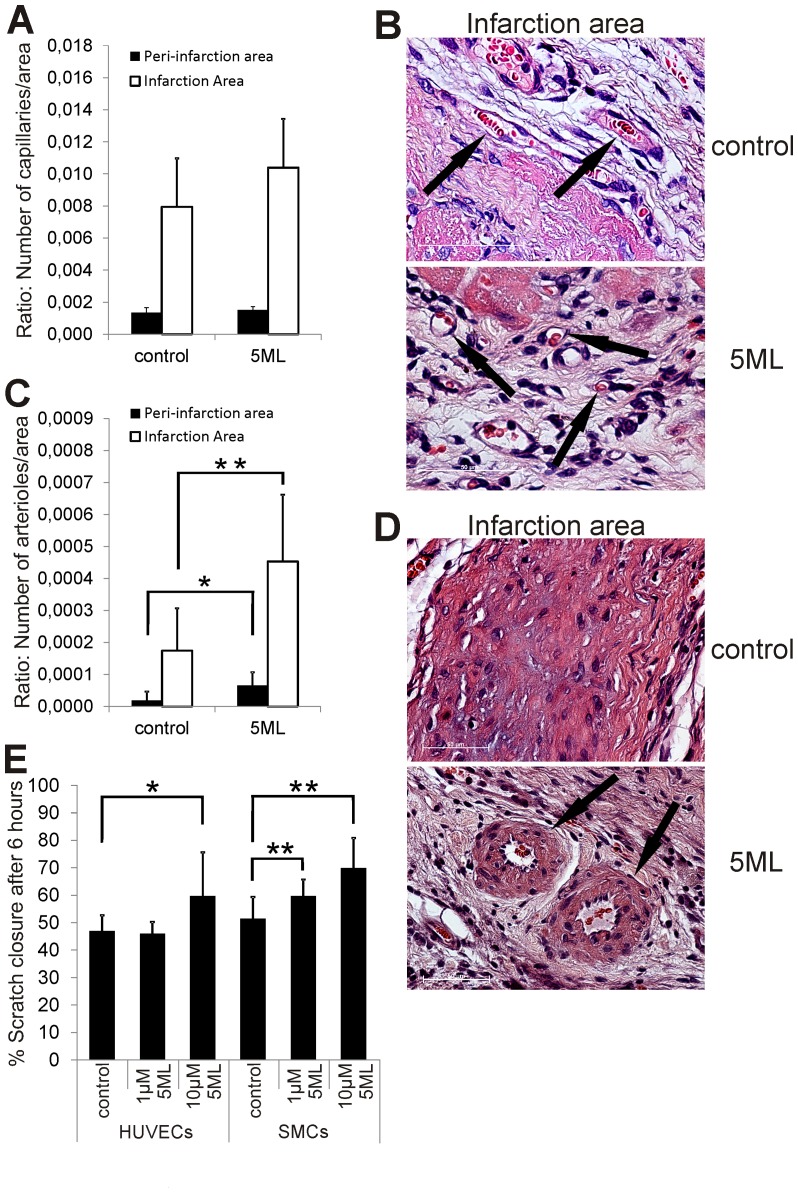
5ML treatment induces arteriogenesis in the peri-infarction and infarction area of rat hearts after MI and activates the migration ability of smooth muscle cells as well as endothelial cells. Figure 6A shows the quantification of the capillary density in the infarction and peri-infarction area on HE stained heart sections (n = 7 per group). Representative images of capillaries in the infraction area are shown in Figure 6B (black arrows; magnification 100×). Quantitative image analysis of the arteriole density in the peri-infarction and infarction area of all animals (n = 7 per group) are shown in Figure 6C and representative images of arterioles in the infarction area are shown in Figure 6D (black arrows; magnification 63×). The effect of 5ML in an *in vitro* wound scratch assay with HUVECs and SMCs is depicted in Figure 6E (data are expressed as% scratch closure after 6 hours). The in vitro experiments were performed in triplicates. Asterisks indicate significant differences (* p<0.05; ** p<0.01) compared to the corresponding controls or control group.

5ML treatment resulted in a significantly increased number of arterioles in the infarction (p = 0.014; Figure 6C and 6D) and peri-infarction area (p = 0.033; Figure 6C) on day 28 post MI. Interestingly, a non-significant trend for increased formation of small arteries in the infarction area in the 5ML group was observed (p = 0.186; Figure S4 in the Supporting Information file). Surprisingly, no significant difference was observed in the number of capillaries in the infarction (p = 0.129; Figure 6A and 6B) and peri-infarction area (p = 1.00; Figure 6A), as well as in Ki67 expression (p = 0.478; data not shown) between the groups. Additional in vitro experiments revealed that 5ML is able to significantly increase the migration ability of endothelial cells (HUVECs) as well as vascular smooth muscle cells (SMCs) (Figure 6E). Analysis of the proliferation of 5ML treated HUVECs and SMCs revealed no effect on smooth muscle cells below 10 µM (significantly reduced proliferation with 10 µM 5ML) but a significant increase in the proliferation of HUVECs treated with 10 µM 5ML (Figure S2A and S2B, Supporting Information file).

## Discussion

To increase angiogenesis, arteriogenesis and therefore the blood supply to tissue is highly desirable in a large number of cardiovascular diseases. Despite some progress that has been made in the past e.g. by applying VEGF and bFGF (including gene therapy), these pro-angiogenic treatments have, until today, not resulted in routine clinical applications. Further, nucleic acids, peptides and proteins are relative large hydrophilic molecules, which significantly limits their diffusion rates, hence their therapeutic effectiveness *in vivo*. Based on this knowledge, we conducted a search for small hydrophobic compounds, capable of stimulating angiogenesis. 5ML, a novel structure type lignan isolated from the roots of Edelweiss, is a potent inducer of angiogenesis *in vitro* and surprisingly, also of arteriogenesis *in vivo*. Based on the results reported herein it seems likely that 5ML stimulates angiogenesis *in vitro* by upregulation of CYP26B1 expression. Previously, several reports have been published showing that an inhibition of CYP activity results in the inhibition of angiogenesis, whereas a stimulation of CYP activity leads to angiogenesis [25], [26]. Reported mechanisms are mainly based on the formation of arachidonic acid metabolites which induce factors like VEGF, MMP9, and EGFR [25], [27]. In line, our experiments show (in the absence of 5ML) that a knock down of CYP1A1 and CYP26B1 potently reduced spontaneous angiogenesis in human endothelial cells (see Figure 3C). Importantly, the increase in angiogenesis by 5ML, was only inhibited by a knock down of CYP26B1, clearly demonstrating the relevance of CYP26B1 in 5ML-induced stimulation of angiogenesis. As can be seen in Figure 3C, 5ML caused only a relatively small increase in HUVEC tube formation. We assume that HUVECs *in vitro* due to potent stimulation by serum and growth factors show a relative high degree of spontaneous tube formation and capillary sprouting, and that for this reason it is hardly possible to increase tube formation and sprouting rates. Yet, 5ML was capable of significantly increasing pro-angiogenic behavior of HUVECs and HMVECs.

In the resting or low proliferating myocardium *in vivo*, the effect of 5ML was significantly higher (Figure 4, 5, and 6). The role of CYP26B1 in angiogenesis has to our knowledge not been studied so far, however CYP26B1 is known to inactivate all-trans-retinoic acid (atRA) by generating hydroxylated forms. atRA is well known to play a significant role in tissue maintenance and differentiation of various cell types, including stem cells [28]. As can be seen in Figure 5C, CYP26B1 expression was nearly absent in the infarct area of control hearts (also the rest of the heart of both groups showed hardly any CYP26B1 expression). Accordingly it may be speculated that the upregulation of CYP26B1 is a physiological response to damage in the heart, aiming to increase vascularisation. Further, we hypothesize that 5ML, by up-regulating CYP26B1 expression and CYP26B1 activity may reduce retinoic acid levels in the infarction area; that reduced retinoic acid levels lead to a reduction of pro-differentiation pressure in the tissue; and that a reduction of differentiation pressure leads to the activation of angiogenesis. However, quantification of capillary and arteriole density in the infarction area revealed an increased number of mature arterioles and no change in the number of capillaries in the 5ML treated hearts. Hence, the authors hypothesize that on day 28 post MI most of the initial immediate regenerative processes like proliferative angiogenesis may no longer be active; but 5ML induced intermediate late processes are on-going, like activated migration of endothelial and smooth muscle cells, needed for the development of arterioles (shown by the *in vitro* wound healing assay).

Surprisingly, in 5ML treated hearts we also observed a significantly higher amount of muscle mass in the infarction area. The origin of these muscle islets is not clear at the moment, however in the absence of increased Ki67 expression (proliferation marker), we assume that 5ML may prevent muscle loss either by i) an accelerated blood supply to the infarct area (increased number of arterioles) or ii) by a direct cardiomyocyte protective activity of the drug during ischemia and reperfusion (protective effect of 5ML is still present in the peri-infarction area). Apart from these concepts, a potential involvement of regeneration processes cannot fully be excluded.

## Conclusion

5ML is one of if not the first low molecular weight compounds with potent pro-angiogenic activity and surprisingly also pro-arteriogenic activity, and potential regeneration stimulatory or cardioprotective properties. Due to its small size (molecular weight 500 Da) and hydrophobic character 5ML has several advantages compared to currently applied pro-angiogenic pharmacons, particularly its higher diffusion rate. Leoligin which has the identical structure as 5ML except for a single methoxy group at 5′ was previously shown to have an half-life in the circulation of several days [29], to exert its drug effect *in vivo* over 2 weeks [16] without any signs of toxicity *in vitro* or in acute and chronic exposure models *in vivo* (unpublished data). By the observation of an increased CYP26B1 expression 28 days after a single application of 5ML, shown in the present work, it could be demonstrated that also 5ML has a long lasting drug effect and potentially also a long half-life, ideal for local treatment of CVDs. Based on these data and currently ongoing toxicity studies it is our goal to extend these studies to large animals and later to potential human trials.

Study Limitations: The core of this project is a combination of in vitro cell culture studies and animal experimentation in rats. As both experimental settings are models for, but cannot replace, studies in humans, the effects observed need further confirmation, in large animals and ultimately in humans. Particularly, the diffusion capacity of the compound 5ML is unclear. In settings of cell culture, diffusion plays a negligible role, and diffusion paths in small animal hearts are much shorter compared to the human heart. Nevertheless, the route and method of application of 5ML may allow solving the problem of limited diffusion. Further, the in vitro signalling pathway analyses are still not complete, and solely identify one central but novel player in angiogenesis induction CYP26B1. Based on this observation we are currently conducting a basic research project, to reveal the entire signalling pathway of 5ML-induced angiogenesis. The results of this study are planned to be confirmed in immunohistological analyses in a large animal model.

In summary, this study provides data that may lead to the development of the first low molecular weight pro-angiogenic and pro-arteriogenic drug. The findings reported herein need to be confirmed and extended in follow up projects.

## Supporting Information

File S1 Figure S1. Overexpression of TXNIP does not interfere with 5-Methoxyleoligin mediated increase in endothelial tube formation. HUVECs stably expressing thioredoxin interacting protein (TXNIP) and controls were incubated with 5ML and subjected to capillary tube formation as outlined in the Material and Methods section. In contrast to CYP1A1 and CYP26B1 knock down, overexpression of TXNIP-which was found to be down-regulated by 5ML had no significant effect on tube spontaneous and 5ML induced formation. Figure S2. 5ML inhibits the proliferation of SMCs and increases the proliferation of HUVECs. To examine the potential effect of 5ML on the proliferation of HUVECs and vascular SMCs in vitro, we performed cellular metabolic assays (XTT) which allows conclusions about the proliferative activity of cells. XTT-based analysis revealed that incubation of HUVECs with 5ML (10 µM) activates significantly the proliferation of the cells, but lower concentrations of 1 µM had no effect. Contrasting results delivered the incubation of vascular smooth muscle cells with 5ML in vitro: concentrations of 1 µM 5ML had no effect on the proliferation. However, incubation with a higher concentration of 10 µM 5ML significantly inhibits the proliferation. Figure S3. 5ML is a stimulator of capillary tube formation with HMVECs. As with HUVECs, 5ML is also able to increase the tube formation with HMVECs (dose dependent increase). Figure S4. 5ML-treated infarction areas show a non-significant increase in the small arteries-density compared to the control hearts. As already mentioned, 5ML is able to significantly increase the number of arterioles in the infarction and peri-infarction area. Further histological analysis revealed there is a non-significant trend towards an increased number of small arteries in the infarction area.
(DOCX)
