# Supplementary material for: 5-Methoxyleoligin, a Lignan from Edelweiss, Stimulates CYP26B1-Dependent Angiogenesis In Vitro and Induces Arteriogenesis in Infarcted Rat Hearts In Vivo
Source: PLoS One. 2013 Mar 12;8(3):e58342. doi: 10.1371/journal.pone.0058342 (PMC3595277; doi:10.1371/journal.pone.0058342)
Supplement: File S1 — Figure S1. Overexpression of TXNIP does not interfere with 5-Methoxyleoligin mediated increase in endothelial tube formation. HUVECs stably expressing thioredoxin interacting protein (TXNIP) and controls were incubated with 5ML and subjected to capillary tube formation as outlined in the Material and Methods section. In contrast to CYP1A1 and CYP26B1 knock down, overexpression of TXNIP-which was found to be down-regulated by 5ML had no significant effect on tube spontaneous and 5ML induced formation. Figure S2. 5ML inhibits the proliferation of SMCs and increases the proliferation of HUVECs. To examine the potential effect of 5ML on the proliferation of HUVECs and vascular SMCs in vitro, we performed cellular metabolic assays (XTT) which allows conclusions about the proliferative activity of cells. XTT-based analysis revealed that incubation of HUVECs with 5ML (10 µM) activates significantly the proliferation of the cells, but lower concentrations of 1 µM had no effect. Contrasting results delivered the incubation of vascular smooth muscle cells with 5ML in vitro: concentrations of 1 µM 5ML had no effect on the proliferation. However, incubation with a higher concentration of 10 µM 5ML significantly inhibits the proliferation. Figure S3. 5ML is a stimulator of capillary tube formation with HMVECs. As with HUVECs, 5ML is also able to increase the tube formation with HMVECs (dose dependent increase). Figure S4. 5ML-treated infarction areas show a non-significant increase in the small arteries-density compared to the control hearts. As already mentioned, 5ML is able to significantly increase the number of arterioles in the infarction and peri-infarction area. Further histological analysis revealed there is a non-significant trend towards an increased number of small arteries in the infarction area. (DOCX) [file pone.0058342.s001.docx]

**SUPPORTING INFORMATION FILE**

5-Methoxyleoligin, a lignan from Edelweiss, Stimulates CYP26B1-Dependent Angiogenesis and Improves Left Ventricular Function of Infarcted Rat Hearts

Barbara Messner, Johann Kern, Dominik Wiedemann, Stefan Schwaiger, Adrian Türkcan, Christian Ploner, Alexander Trockenbacher, Klaus Aumayer, Nikolaos Bonaros, Günther Laufer, Hermann Stuppner, Gerold Untergasser, and David Bernhard.

**METHODS:**

**Generation of HUVEC derivatives with constitutive TXNIP expression**

The lentiviral constitutive expression constructs pHR-PGK-TXNIP-bGh-polyA-SFFV-Puro (U557), pHR-PGK-3FLAG TXNIP-bGh-polyA-SFFV-puro (U561) were generated using the GATEWAY^TM^ technology (Invitrogen, Carlsbad, CA). The details of this procedure and the generation of stably transduced bulk populations of cells with constitutive expression of cDNAs cloned into such constructs has been described previously [1] In brief, human TXNIP mRNA was PCR-amplified from IRATp970D06117D (Invitrogen) using forward primer 5`-CAAAAAAGCAGGCTCCATGGTGATGTTCAAGAAGATCAAGTC-3` and reverse primer 5`-CAAGAAAGCTGGGTCTCACTGCACATTGTTGTTGAGG-3`or using forward primer 5`- CaaaaaagcaggctccATGGACTACAAAGACCATGACGGTGATTATAAAGATCATGACATCGATTACAAGGATGACGATGACAAGgtgatgttcaagaagatcaagtc-3` and reverse primer 5`-CAAGAAAGCTGGGTCTCACTGCACATTGTTGTTGAGG-3`(for introduction of a N-terminal 3xFLAG tag to TXNIP).

The PCR products (after a second PCR reaction to complete the attB sites) were recombined into pDONR207 (Invitrogen) resulting in pENTR207-TXNIP

(U558), pENTR207-3FLAG-TXNIP (U562). All pENTR 207 constructs were sequence-verified and subsequently recombined into the “destination vector” pHR-PGK-DEST-bGh-polyA-SFFV-Puro (U538, Ploner C. et.al. in preparation) to generate pHR-PGK-TXNIP-bGh-polyA-SFFV-Puro (U557); pHR-PGK-3FLAG TXNIP-bGh-polyA-SFFV-Puro U561 respectively.

Generation of stably transduced bulk populations of cells with constitutive expression of cDNAs was described elsewhere {Ploner et al. [1]}. In short, human HEK 293T cells were transiently transfected with lentiviral plasmids U557, U559 or U561 along with the packaging plasmids pSPAX and pVSV-G (kindly provided by Didier Trono). Forty eight and 72 hours after transfection lentiviruscontaining supernatant was sterile filtered (0.2µM) and concentrated using poly-ethylene glycol [2]; concentrated virus was supplemented with polybrene to a final concentration of 4µg/ml and used to transduce HUVEC cells.

**Cell culture**

Human microvascular endothelial cells (MVECs) were purchased from Promocell (Vienna, Austria) and cultured as previously described [3, 4].

**Analysis of the number of viable cells**

The number of viable cells (HUVECs and vascular SMCs) was determined using the XTT assay (Biomol; Hamburg, Germany) according the manufacturer´s instructions. HUVECs and smooth muscle cells were seeded into gelatin coated 96well plates and allowed to adhere overnight. The next day, the medium was replaced by fresh medium and the cells were treated with the indicated 5ML concentrations and DMSO as solvent control. The number of viable cells was assessed after 24, 48 and 72 hours of treatment. The reduction of the tetrazolium salt XTT was measured at 450nm (reference wavelength: 595nm) using a Victor3 microplate reader (Victor 3, Perkin Ellmer; Austria).

**Capillary tube formation assay**

To analyze capillary tube-formation, 24-well-plates were coated with 200 µl growth factor reduced Matrigel (BD Biosciences). MVECs (5 x 10^4^ cells) were resuspended in 200 µl EGM-2 medium with or without 5-Methoxyleoligin (1 µM, 10 µM) and placed on the polymerized matrix, followed by the analysis of tube formation after 6 h. Tubes were visualized by an inverted transmission-microscope (Zeiss Axiovert 200M) and documented by a digital imaging system (Axiovision Software, Zeiss). Statistical analysis was performed after calculating capillaries/mm.

**Immunohistological and histological analyses**

Following fixation in 4% paraformaldehyde and dehydration of heart tissues, tissues were embedded in paraffin and cross sections were prepared. After deparaffinization, Hämatoxylin/eosin (HE) stainings were performed according to the manufacturers´ instructions to analyze and count the number of small arteries (identified as arteries with 3-8 layers of smooth muscle cells) in the defined infarction. Image analysis was conducted by two independent blinded researchers.

**RESULTS:**

**Overexpression of TXNIP does not interfere with 5-Methoxyleoligin mediated increase in endothelial tube formation**

HUVECs stably expressing thioredoxin interacting protein (TXNIP) and controls were incubated with 5ML and subjected to capillary tube formation as outlined in the Material and Methods section. In contrast to CYP1A1 and CYP26B1 knock down, overexpression of TXNIP – which was found to be down-regulated by 5ML – had no significant effect on tube spontaneous and 5ML induced formation.

**Supporting Information Figure S1:**

Supporting Information Figure S1 shows that TXNIP expression in HUVECs has neither a significant effect on spontaneous nor 5ML-induced angiogenesis in HUVECs. Shown are mean values of two independent experiments performed in triplicates +/- SD.

**5ML inhibits the proliferation of SMCs and increases the proliferation of HUVECs.**

To examine the potential effect of 5ML on the proliferation of HUVECs and vascular SMCs in vitro, we performed cellular metabolic assays (XTT) which allows conclusions about the proliferative activity of cells. XTT-based analysis revealed that incubation of HUVECs with 5ML (10µM) activates significantly the proliferation of the cells, but lower concentrations of 1µM had no effect. Contrasting results delivered the incubation of vascular smooth muscle cells with 5ML in vitro: concentrations of 1µM 5ML had no effect on the proliferation. However, incubation with a higher concentration of 10µM 5ML significantly inhibits the proliferation.

**Supporting Information** **Figure S2:**

Supporting Information Figure S2: Figure S2A shows that 1µM 5ML has no effect on the proliferation of vascular smooth muscle cells, whereas treatment with 10µM 5ML significantly inhibits the proliferation of these cells. Figure S2B: Further, 1µM 5ML has no significant effect on the proliferation of HUVECs, but 10µM 5ML significantly increases the proliferation of HUVECs.

**5ML is a stimulator of capillary tube formation with HMVECs.**

As with HUVECs, 5ML is also able to increase the tube formation with HMVECs (dose dependent increase; Supporting information Figure S3).

**Supporting Information Figure S3:**

Supporting Information Figure S3 shows the effect of 5ML treatment on HMVECs in a capillary tube formation assay.

**5ML-treated infarction areas shows a non-significant increase in the small arteries-density compared to the control hearts.**

As already mentioned, 5ML is able to significantly increase the number of arterioles in the infarction and peri-infarction area. Further histological analysis revealed there is a non-significant trend towards an increased number of small arteries in the infarction area.

**Supporting Information Figure S4:**

Supporting Information Figure S4 shows the number of small arteries (3-8 layers of smooth muscle cells) in the defined infarction area.

**REFERENCES**

[1] Ploner C, Rainer J, Niederegger H, Eduardoff M, Villunger A, Geley S, Kofler R: The BCL2 rheostat in glucocorticoid-induced apoptosis of acute lymphoblastic leukemia. *Leukemia* 2008, 22:370-377.

[2] R.H. Kutner, X.Y. Zhang, J. Reiser, Production, concentration and titration of

pseudotyped HIV-1-based lentiviral vectors, Nat. Protoc. 4 (2009) 495–505.

[3] Messner B, Knoflach M, Seubert A, Ritsch A, Pfaller K, et al. (2009) Cadmium is a novel and independent risk factor for early atherosclerosis mechanisms and in vivo relevance. Arterioscler Thromb Vasc Biol 29: 1392-1398.

[4] Reisinger U, Schwaiger S, Zeller I, Messner B, Stigler R, et al. (2009) Leoligin, the major lignan from Edelweiss, inhibits intimal hyperplasia of venous bypass grafts. Cardiovasc Res 82: 542-549.
